# Implementation and Evolution of Mitigation Measures, Testing, and Contact Tracing in the National Football League, August 9–November 21, 2020

**DOI:** 10.15585/mmwr.mm7004e2

**Published:** 2021-01-29

**Authors:** Christina D. Mack, Erin B. Wasserman, Cria G. Perrine, Adam MacNeil, Deverick J. Anderson, Emily Myers, Sabrina Smith, L. Clifford McDonald, Michael Osterholm, Gary S. Solomon, Thom Mayer, Allen Sills, NFL COVID-19 Advisory and Operational Team, NFL COVID-19 Advisory and Operational Team, Dawn Aponte, Michele Best, Paul Blalock, Meghan C. Carroll, M. Anthony Casolaro, Molly Delaney, Daniel Eichner, Larry Ferazani, Jacob Frank, Christopher J. Hostler, Tiffany Koch, John Lynch, Jimmie Mancell, Damion Martins, John Mellody, Jeff Miller, Navdeep Singh, Eric Sugarman, Leah Triola, Patti Walton

**Affiliations:** ^1^IQVIA Real-World Solutions, Research Triangle Park, North Carolina; ^2^CDC COVID-19 Response Team; ^3^Department of Medicine, Duke Center for Antimicrobial Stewardship and Infection Prevention, Durham, North Carolina; ^4^National Football League, New York, New York; ^5^Center for Infectious Disease Research and Policy, University of Minnesota, Minneapolis, Minnesota; ^6^National Football League Players Association, Washington, D.C.; National Football League; University of Maryland Capital Region Health; National Football League; National Football League; Washington Football Team; National Football League; Sports Medicine Research and Testing Laboratory; National Football League; National Football League; Department of Medicine, Duke Center for Antimicrobial Stewardship and Infection Prevention; IQVIA Real-World Solutions; Department of Medicine, University of Washington; Department of Medicine, University of Tennessee Health Science Center; Atlantic Sports Health, Morristown Medical Center; National Football League; National Football League; Eden Medical Center; Minnesota Vikings; National Football League; Williamson Medical Center.

The National Football League (NFL) and the NFL Players Association (NFLPA) began the 2020 football season in July, implementing extensive mitigation and surveillance measures in facilities and during travel and gameplay. Mitigation protocols* were evaluated and modified based on data from routine reverse transcription–polymerase chain reaction (RT-PCR) tests for SARS-CoV-2, the virus that causes coronavirus 2019 (COVID-19); proximity tracking devices; and detailed interviews. Midseason, transmission was observed in persons who had cumulative interactions of <15 minutes’ duration, leading to a revised definition of high-risk contacts that required consideration of mask use, setting and room ventilation in addition to proximity and duration of interaction. The NFL also developed an intensive protocol that imposed stricter infection prevention precautions when a case was identified at an NFL club. The intensive protocol effectively prevented the occurrence of high-risk interactions, with no high-risk contacts identified for 71% of traced cases at clubs under the intensive protocol. The incorporation of the nature and location of the interaction, including mask use, indoor versus outdoor setting, and ventilation, in addition to proximity and duration, likely improved identification of exposed persons at higher risk for SARS-CoV-2 infection. Quarantine of these persons, along with testing and intensive protocols, can reduce spread of infection.

The NFL consists of 32 member clubs based in 24 states. The NFL-NFLPA implemented a standard COVID-19 mitigation protocol in July that included mandatory masking; physical distancing; frequent handwashing; facility disinfection; restricted facility access; and regular, frequent testing of players and staff members (*1*). Contact tracing was performed by trained staff members and supported by KINEXON wearable proximity devices (https://kinexon.com) that were required to be worn by players and personnel when in club environments (*2*). Device recordings captured consecutive and cumulative minutes/seconds of interactions among persons within 1.8 meters (6 feet) of one another. When testing identified a new COVID-19 case, trained staff members conducted interviews to identify contacts including and beyond device-identified persons (e.g., nonclub activities, social interactions, and times when the device was not worn). RT-PCR tests, with results available in 24 hours, were initially conducted 6 days per week for players and most staff members.^†^ Analyses were performed to actively evaluate the efficacy of the NFL-NFLPA protocols in limiting high-risk interactions and preventing COVID-19, including comprehensive review of RT-PCR results, device-recorded interactions, and contact tracing interviews. This activity was reviewed by CDC and was conducted consistent with applicable federal law, CDC, and NFL-NFLPA policy.^§^

Over the course of the monitoring period (August 9–November 21), 623,000 RT-PCR tests were performed among approximately 11,400 players and staff members; 329 (approximately 2.9%) laboratory-confirmed cases of COVID-19 were identified. After intake screening,^¶^ in August and early September, fewer than 10 COVID-19 cases were identified per week for the following 7 weeks (Figure), during which time the standard protocol was in effect, which emphasized physical distancing, masking, limited numbers of persons in specific areas, and other important behavioral and facility-related parameters. However, during September 27–October 10, a total of 41 cases were identified among players and staff members, 21 of which were believed to have resulted from within-club transmission at a single club, requiring closure of that club’s facilities. Subsequent contact tracing identified multiple instances of transmission that likely occurred during <15 minutes of cumulative interaction within 1.8 meters (6 feet). Among the 21 persons with suspected within-club transmission, 12 had no device-recorded interactions of ≥15 consecutive minutes with a person with confirmed COVID-19, including eight who had no interactions >5 consecutive minutes and seven who had no interactions >15 cumulative minutes per day (with no other known exposures to a person with COVID-19). Interviews revealed that, among the brief interactions that did occur, some were during unmasked meetings in small rooms or while eating. Persons who contracted COVID-19 within this single-club transmission group received negative test results for several days after exposure (i.e., after club activities ceased) before receiving a positive result.

**FIGURE:**
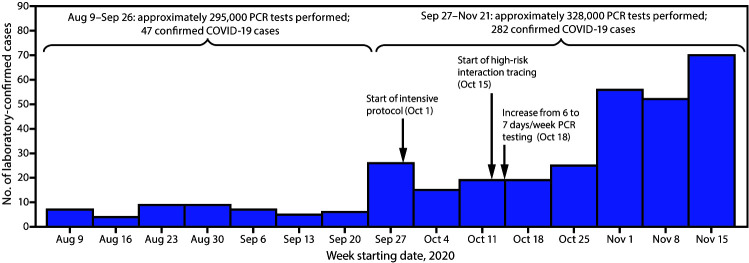
Laboratory-confirmed* COVID-19 cases (N = 329) and mitigation strategies^†^ implemented — National Football League, United States, August 9–November 21, 2020 **Abbreviations:** COVID-19 = coronavirus disease 2019; PCR = polymerase chain reaction. * Reverse-transcription PCR tests were processed on two platforms (Roche Cobas and ThermoFisher QuantStudio) and transcription-mediated amplification on one platform (Hologic Panther Aptima). ^†^ Twenty-nine clubs spent 431 days under the intensive protocol beginning October 1; 189 high-risk contacts of 215 cases were identified and subsequently quarantined beginning October 15.

After this cluster of cases, several league-wide changes were implemented. The first involved the clubs moving to an intensive protocol for 7 days when a positive test result was received; the intensive protocol mandated further restrictions for the entire club to mitigate spread (Table 1). The intensive protocol was implemented for any club if any players or staff members with facility access contracted COVID-19, or if the team played a game against an opposing player who received a next-day positive result from his game-day test. During October 1–November 21, among the 32 clubs, 29 spent 431 days under the intensive protocol. During this time, the median number of within-facility interactions of ≥15 consecutive minutes at <1.8 meters (<6 feet) per club per day decreased by 60%, from 60 to 24, and interactions of ≥2 consecutive minutes decreased by 28%, from 1,691 to 1,222. The second change involved increasing testing frequency from 6 to 7 days per week. A third league-wide change was expansion of contact tracing and transmission risk assessment focusing on high-risk contact identification, which comprised four main components. These were, in addition to consideration of duration of exposure and specific distance between persons, assessment of face mask use (e.g., medical mask versus cloth face covering, proper mask use for both infected person and contact, and any mask removal to eat or drink) and setting and ventilation (e.g., outdoor, indoor large volume, indoor small volume, and during transportation).** Expanded contact tracing covered all club-related contacts of persons with confirmed COVID-19 within the preceding 48 hours, including those outside the facility, with interviews regarding the full context of exposure and medical expert evaluation of the risk level for each interaction. Designation of a high-risk contact generally required concern by medical experts about the interaction involving two or more components; mask use and outdoor settings were considered protective. For example, short car rides with partial mask use were considered high-risk, whereas prolonged interaction (>15 minutes) in well-ventilated settings (e.g., outdoors) with proper mask use were not. Contact tracing interviews and adjudication of high-risk contact status were typically completed within 18 hours of a positive test result. All contacts of COVID-19 patients, regardless of duration of interaction, were instructed to remain out of club facilities until high-risk status determination was complete. Persons could also be designated high-risk contacts if a household member received a positive test result (*3*); self-reporting of cases among household members was required. The mandatory minimum quarantine for high-risk contacts was 5 days postexposure, shorter than that recommended in CDC guidance (*4*); this was deemed acceptable because daily RT-PCR testing with <24-hour turnaround was available. Upon release from quarantine, high-risk contacts continued daily testing and symptom monitoring, enabling rapid identification and isolation of persons who received positive test results after quarantine.

**TABLE 1:** Summary of standard and intensive COVID-19 mitigation protocols — National Football League/National Football League Players Association, United States, August–November 2020

Standard protocol	Intensive protocol (modifications/stipulations)*
**Meetings**	
Conducted virtually to the extent possible	All meetings must be held virtually
If in-person meetings are necessary, clubs must make efforts to conduct them outdoors with physical distancing and masking	If in-person necessary, meetings must be held outdoors or in large domed (tented) practice field with physical distancing, masking, and with all attendees wearing proximity tracking devices, with specific approval by medical experts
In-person meetings without physical distancing prohibited	
Meetings with >15 persons must be virtual, unless physical distancing is possible	
**Practice/Walkthrough**	
All staff members must wear masks at all times on the practice field, and all players must wear masks on the practice field when feasible; surgical masks preferred, and gaiters, valved/vented masks prohibited	All players must wear masks or Oakley face shield during practices at all times without exception
	Staff members must wear masks at all times; gaiters, valved/vented masks prohibited
Masks mandatory during walkthroughs	Players may remove helmets/masks for breaks but must maintain >6 feet (1.8 meters) of distance
**Weight room**	
Maximum capacity of 15 players (no limit on staff members)	Maximum capacity of 10 players and five staff members
Must maintain 6 feet (1.8 meters) of distance	Further emphasis on appropriate distancing
All staff members must wear masks	Players and staff members must wear masks at all times
Players encouraged to wear masks but not mandatory	
**Medical treatment/rehabilitation**	
Masks required during medical treatment and rehabilitation inside the club facility; surgical masks preferred	Players must wear a surgical grade mask at all times and a face shield when possible
	Staff members must wear a face shield and surgical grade mask and gloves at all times
**RT-PCR testing**	
Negative RT-PCR test result from the previous day before facility entrance is permitted	RT-PCR test results returned for all players from previous day before any players or staff members are permitted in the facility (negative result required for entry)
**Cafeteria/Meal area**	
Meal room access limited	No seating permitted in cafeteria or meal area (grab-and-go only)
Tables distanced to allow for 10 feet between persons while consuming food and drink	
Clubs expected to discourage group dining	
Clubs expected to stagger mealtimes	
Whenever possible, premade meals should be provided in individually packaged containers or bags for takeout	
Disposable utensils, plates and single-use condiments must be used	
Buffet-style, communal and self-serve spreads prohibited	
**Locker rooms**	
Locker room reconfigured to allow for 6 feet (1.8 meters) between players; if not possible, clubs must consider other measures (e.g., plexiglass dividers between lockers and temporary lockers in tented areas)	Locker room use strongly discouraged
Minimize time players spend in locker room	Use must be <15 minutes per person per session
Minimize number of players in the locker room	Limited to smaller groups
Masks required at all times, except in the shower	
**Gatherings**	
Groups of more than three persons prohibited from gathering outside of facility or team travel	No in-person contacts among players or essential staff members outside of facility or team travel

**Abbreviations:** COVID-19 = coronavirus disease 2019; RT-PCR = reverse transcription–polymerase chain reaction.

*The intensive protocol includes all the components of the standard protocol, with modifications or stipulations listed.

During October 15–November 21, a total of 189 NFL players and staff members were identified as high-risk contacts of 215 persons with confirmed COVID-19 and were subsequently quarantined. Among these, 20 (11%) persons from 12 clubs received positive test results (mean and median interval from exposure to positive RT-PCR sample collection = 5 days [range = 1–9 days]) (Table 2). Seven of these 20 contacts received positive test results after release from 5-day quarantine; however, they continued to test daily and adhere to strict mitigation measures, and no within-club secondary transmission was identified among these persons. Among those exposed outside of the home, all reported partial or no mask use, and the majority of exposures were external to the NFL environment (e.g., sharing a vehicle and eating at a restaurant). Among 107 traced cases among clubs already in the intensive protocol at the time of positive test result, 76 persons (71%) had no high-risk contacts identified.

**TABLE 2:** Characteristics of high-risk interactions* between persons who were identified as high-risk contacts of a COVID-19 patient, quarantined, and subsequently received a positive SARS-CoV-2 test result (N = 20) — National Football League (NFL), United States, October 15–November 21, 2020

Characteristic	No.^†^
**Total contacts**	**20**
**Household contacts (family member or roommate)^§^**	8
**Nonhousehold contacts** ^¶^	12
** Work environment**	4
Within 1.8 m (6 ft)**	2
>15 cumulative minutes of contact	4
No facial covering or partial facial covering	4
Indoors (club facility/hotel^††^)	4
Involved dining	4
** Nonwork environment**	8
Within 1.8 m (6 ft)	8
>15 cumulative minutes of contact	7
No or partial face covering	8
Indoors^§§^	8
Involved dining^¶¶^	5

**Abbreviation:** COVID-19 = coronavirus disease 2019.

* Identified through expanded contact tracing. The 20 high-risk contacts were from 12 clubs.

^†^ If information about the specific interaction was unknown, person was excluded.

^§^ Two of the eight household high-risk contacts worked and lived with the index person with COVID-19 and likely also had work environment interactions but are excluded from that analysis because of the high-risk level of household exposures.

^¶^ Based on KINEXON device data and interviews (n = 4) or interviews only (n = 8). Multiple interactions are possible.

**Unknown for two of four.

^††^ Three of the four were exposed in a team hotel; one of the four was exposed in an NFL club facility.

^§§^ Five of the eight were exposed at a party/social gathering, one of the eight in a restaurant, and two of the eight in a car.

^¶¶^ Unknown for one contact.

## Discussion

Real-time evaluation of surveillance data and response to suspected COVID-19 transmission events within NFL clubs led to important changes in NFL-NFLPA COVID-19 protocols. Compulsory 7-day intensive protocol implementation for clubs with any exposure to COVID-19, mandatory 5-day quarantine of high-risk contacts, and daily RT-PCR testing effectively reduced exposure and facilitated earlier case identification. Daily testing allowed early, albeit not immediate, identification of infection (*5*), necessitating quarantine after exposure; high frequency testing also facilitated real-time program evaluation.

To date, the ability to define a close contact has been limited. An investigation from a Vermont corrections facility confirmed that cumulative brief interactions exceeding 15 minutes in total could lead to transmission (*6*). However, among 21 NFL cases for which contact tracing indicated likely within-club transmission, seven infected persons had no interactions exceeding 15 cumulative minutes per day within 1.8 meters (6 feet) of a person with COVID-19, as confirmed by wearable proximity devices. This finding led to a revised high-risk contact definition that included ascertainment of mask use and setting, in addition to duration of exposure and proximity.

Although proximity devices provided detailed information about possible high-risk interactions, prompt, detailed, contact tracing beyond proximity device data was needed to identify high-risk behaviors and enable quarantine of exposed persons. All high-risk contacts who subsequently received a COVID-19 diagnosis were identified, at least in part, from information obtained through interviews. Indoor unmasked activities, ridesharing in personal vehicles, and eating and drinking in close proximity were of particular risk, as has been previously reported (*7*).

An intensive protocol designed for this environment and deployed to facilities with known exposure was an effective mitigation measure. Some NFL clubs chose to retain intensive protocol restrictions beyond mandatory periods; implementation and completion of an intensive protocol can serve an important motivator and reminder of the need for diligence (*8*). The quarantine of exposed persons and ability of the full employee population to move into a more restrictive protocol during periods of increased risk is an intervention that could be extended to settings such as long-term care facilities, schools, and high-density environments (*9*). The intensive protocol was likely critical in preventing transmission of SARS-CoV-2 because seven of 20 quarantined high-risk contacts did not receive a positive test result until completing their 5-day quarantine. In scenarios without daily testing, duration of both quarantine and intensive protocol implementation might require extension. Intensive protocol restrictions can be tailored to each environment to include, at minimum, more extensive masking and outdoor venue use and further restrictions in access, room volume, in-person meetings, and mealtime interactions.

The increase in cases identified in NFL clubs in October and November mirrored the increased incidence in the United States during that time (*10*). These infections were primarily related to community exposures, based on contact tracing interviews and exemplified by the high proportion of persons who contracted COVID-19 after household exposure. Although the intensive protocol and high-risk contact designations were primarily intended to prevent work-related exposures, employees were regularly educated about risks from household and community exposure. Implementation of the intensive protocol decreased within-facility exposures despite increasing community transmission of COVID-19 across the country during this time.

The findings in this report are subject to at least three limitations. First, wearable device metrics rely on adherence; individual-level compliance is unknown. Second, determination of high-risk contact status is interview-based and subject to recall and reporting bias; household exposures are based on self-report. Finally, source and date of transmission cannot be confirmed.

COVID-19 mitigation measures must be continually optimized based on available data. In the NFL, COVID-19 transmission was identified in persons with <15 minutes of consecutive or cumulative interaction and was reduced through implementation of an intensive protocol focused on environmental change, increased personal protection, avoidance of high-risk interactions such as vehicle sharing, eating in the same room or common areas, and expansion of the components of contact tracing to incorporate high-risk contact designations. Although the protocols implemented by the NFL were resource-intensive, strategies such as accounting for specific characteristics of the close contact, in addition to time and duration, and creation of an intensive protocol are applicable to other settings, including essential workplaces, long-term care facilities, and schools.

**Summary**

**What is already known about this topic?**

COVID-19 contact tracing is important to prevent transmission, but risk characterization is difficult.

**What is added by this report?**

The National Football League observed SARS-CoV-2 transmission after <15 minutes of cumulative interaction, leading to a revised definition of a high-risk contact that evaluated mask use and ventilation in addition to duration and proximity of interaction. Intensive mitigation protocols effectively reduced close interactions.

**What are the implications for public health practice?**

Assessment of the context of each interaction, including mask use, indoor versus outdoor setting, and ventilation, in addition to duration and proximity, can improve identification of high-risk contacts during contact tracing. Postexposure quarantine based on redefined high-risk criteria, combined with testing and environment-specific intensive protocols, can protect communities before and after case identification.

## Footnotes

*
https://www.playsmartplaysafe.com/wp-content/uploads/2020/10/nfl-nflpa-covid-protocols-updated-10.16.20-final.pdf

† A single lab provider with five geographically dispersed laboratories, BioReference Laboratories, provided <24-hour turnaround nucleic acid amplification testing, with Roche Cobas, Hologic Panther, and ThermoFisher QuantStudio as the primary molecular platforms. Tests were administered via anterior nasal swabs. Staff members whose job functions required regular direct access to players for >10 minutes at a time and those who would regularly be in close proximity to players were tested 6 days per week (approximately two thirds of staff members). Consultants who were only in contact with players on a periodic basis were tested less frequently and immediately before access to players and club personnel. Other staff members who performed facility, stadium, or event services but did not require close contact with other persons were tested once per week.

§ 45 C.F.R. part 46.102(l)(2), 21 C.F.R. part 56; 42 U.S.C. Sect. 241(d); 5 U.S.C. Sect, 552a; 44 U.S.C. Sect. 3501 et seq; March 15, 2020 NFL-NFLPA Collective Bargaining Agreement, Article 39, Section 18, Appendix X.

¶ Before reporting to the club facility for preentry testing, players and staff members completed a symptom and exposure questionnaire. Staff members were tested via RT-PCR test nasal swab on two occasions separated by 72 hours. After two negative tests, entry into the facility was allowed and daily testing began. Players were RT-PCR tested on day 1, day 2, and day 4. If players had negative test results on days 1, 2, and 4, they were allowed to enter the facility with daily RT-PCR testing beginning on day 5. Quarantine was required during intake testing. Daily symptom screening continued through intake testing and throughout the monitoring period. Antibody testing was offered but not required and did not impact behavioral requirements mandated by the NFL-NFLPA COVID-19 protocols.

** Modified from https://english.elpais.com/society/2020-10-28/a-room-a-bar-and-a-class-how-the-coronavirus-is-spread-through-the-air.html. Accessed November 20, 2020.
